# MUSCLE PRESERVATION AND PHYSICAL ACTIVITY: HYBRID TRANSCRIPTOMIC ANALYSIS IN A MONKEY MODEL

**DOI:** 10.1093/geroni/igae098.2631

**Published:** 2024-12-31

**Authors:** Stefano Donega, Nirad Banskota, Supriyo De, Luigi Ferrucci

**Affiliations:** National Institute on Aging, Baltimore, Maryland, United States; National Institutes of Health, Bethesda, Maryland, United States; National Institute on Aging, Baltimore, Maryland, United States; National Institute on Aging, Baltimore, Maryland, United States

## Abstract

Muscle strength declines with age, leading to decreased mobility. Old sedentary people have been reported with imbalance in energetic metabolism, a pro-inflammatory profile, and proteolysis. Engaging in exercise, beyond just strength training, has been shown to mitigate this decline, although the underlying biological mechanisms remain unclear. Rhesus monkeys, trained to run on treadmills, present a valuable model for investigating the mechanisms involved in the preservation of muscle function induced by physical activity. We trained 27 males and females monkeys aged 9-15 years for 2 month to run on a ramp up to 1h for 3-5 days/week for 3 months. Continuous monitoring of heart rate, temperature, and physical activity levels was conducted using implantable data loggers, along with daily tracking of body weight and food intake. Vastus lateralis muscle punches were collected at baseline, after 3-months treadmill and ensuing 3-week sedentary state to perform deep hybrid RNA-seq combining deep short-reads (800M, Illumina) and long-reads (PacBio). Treadmill intervention revealed an enrichment in MAPK activity, keratinocyte proliferation, extracellular transport and microtubule formation alongside reductions in ribosomal compartments, ncRNA processing and lysosome. Upon transitioning to a sedentary state, the same subjects increased in extracellular matrix, immune cells migration and chemotaxis (leukocytes, granulocytes, neutrophils and monocytes) and reduced mitochondrial component and function, spliceosome, U2 complex, and ribosome. On-going analyses are focusing on differential splicing and proteomics interaction. These preliminary data suggest at RNA level, exercise enriches muscle function and metabolism while sedentary leads to decreased mitochondrial function and immune cell dynamics in monkeys.

